# Drug Metabolism and Pharmacokinetics

**Published:** 2008-10

**193**

**Effect of esomeprazole on pharmacokinetic of phenytoin**

Prasad Byrav DS, Medhi B, A Prakash, Joshi Rupa

***Postgraduate Institute of Medical Education and Research, Chandigarh, India.***

Esomeprazole is presently most commonly prescribed protom pump inhibitor for gastritis and peptic ulcer. In clinical practice, phenytoin and esomeprazole are prescribed in a chronic condition of generalized seizure with concomitant peptic ulcer. Hence there are chances of drug-drug interaction because of modulations of isoenzymes CYP2C9/10 and CYP2C19 are involved in metabolism of phenytoin and esomeprazole. It is important to maintain the therapeutic level of phenytoin in plasma for effective control of seizure. So, the aim of the study was to determine the effect of esomeprazole on the pharmacokinetics of phenytoin in rabbits. In a parallel design study, phenytoin 30 mg/kg/day per oral was given daily for 14 days. On day 15, blood samples were taken at various time intervals between 0-24 hours. In esomeprazole group, phenytoin was administered for seven days as above. On day 8, esomeprazole 2.8 mg/kg along with phenytoin 30 mg/kg/day was administered and blood samples drawn as above. Plasma phenytoin levels were assayed by HPLC and pharmacokinetic parameters were calculated. In esomeprazole- phenytoin group, there was significant increase of t½ el than phenytoin alone group and significant changes were observed in the pharmacokinetic parameters in esomeprazole treated group. These results suggest that esomeprazole alter the pharmacokinetics of phenytoin. Confirmation of these results in clinical studies will warrant changes in phenytoin dose or frequency when esomeprazole is co-administered with it.

**194**

A novel study of mass spectrometric fragmentation of adrafinil, modafinil and their metabolite modafilinic acid under ESI-LC-MS/MS and EI-GC-MS

Dubey S, Ahi S, Beotra A, Reddy IM, Kaur T, Jain S

***National Dope Testing Laboratory, Ministry of Youth affairs and Sports, New Delhi, India.***

**Introduction:**

Adrafinil and Modafinil are clinically used in the treatment of narcolepsy, obstructive sleep apnea and idiopathic hypersomnia. However, use of these drugs is banned in sports by WADA due to their Central Nervous system stimulating effects. Traditionally, these drugs are detected as a single peak by Gas Chromatographic- Mass Spectrometric (GC-MS) analysis. The objective of the present paper is to explore the possibility of differentiation of Modafinil, Adrafinil and Modafinilic Acid by Liquid Chromatographic-Tandem Mass spectrometric (LC-MS/ MS) technique which is not possible be GC-MSD.

**Materials and Methods:**

The urine samples spiked in 4 replicates with Modafinil and Adrafinil in different concentration were processed by two different methods and injected into GC-MS and LC-MS/MS. The excretion study samples were also processed in the same way and injected. The data were analyzed by comparing the mass spectrum obtained by the above-mentioned techniques.

**Results:**

The results show that Modafinil, Adrafinil and their major metabolite Modafilinic acid could be detected as a single artifact without differentiation in EI-GC-MS analysis whereas the above three could be detected as well as differentiated in ESI-LC-MS/MS analysis. The method was validated and applied for excretion study samples generated by giving one single dose tablet of Modafinil (100 mg, Sun pharmaceuticals, India) to a male healthy volunteer. The sample shows the presence of the parent drug Modafinil and the primary metabolite Modafinilic acid.

**Conclusions:**

The detection procedure using ESI-LC-MS/MS allows the confirmation of Modafinil, adrafinil and Modafinilc acid. This procedure may be used for confirmation of suspicious samples found in routine testing on GC-MSD.

**195**

**Pharmacokinetics of single oral dose of ofloxacin 200 mg in healthy volunteers**

Shakya R^1^, Hada M^1^, Thapa P^1^, Saha RN^2^

***^1^Kathmandu University, Kavre, Nepal; ^2^B.I.T.S., Pilani, Rajasthan, India.***

Ofloxacin is a widely used fluoroquinolone with broad anti-microbial spectrum. Its pharmacokinetic information is necessary for adjusting the dose to achieve MIC. This study evaluated the pharmacokinetics of ofloxacin following oral administration. 8 healthy adult males received a single dose of 200 mg ofloxacin tablet after a standard breakfast. Blood samples were collected at 0, 0.25. 0.5, 0.75, 1, 2, 3, 4, 6, 8, 12, 16 and 24 hours of dosing. For determination of drug concentration in serum, a simple, isocratic HPLC method was developed and validated. Separation was performed on Capcell C_18_ column (250 mm × 4.6 mm ID, 5 *µ*m) using a mobile phase of acetonitrile with 0.0625% triethylamine in water (12.5:87.5 v/v, pH 2.5) at a flow rate of 1.2 ml.min^−1^. Methanol was used for protein precipitation. Subjects were monitored carefully for side effects. All of them completed the study without drug-related problems except one who experienced minor side effects like nausea, headache and palpitation. Pharmacokinetic parameters were calculated on the basis of open two compartmental model. It was found that following oral administration, there is rapid and extensive absorption from gastrointestinal tract achieving mean peak serum concentration of 1.81 mcg.ml^−1^ at 1.92 hours. The terminal elimination half life of ofloxacin was found to be 5.01 hours and elimination rate constant was 0.13 hour^−1^. The value of AUC_0-t_ and AUC_0-inf_ was found to be 13.51mcg.hr.ml^−1^ and 14.03 mcg.hr.ml^−1^ respectively. Hence, pharmacokinetic profile after administration of 200mg tablet of ofloxacin seems to be optimum to achieve MIC for several microorganisms.

**196**

**Utility of LC-MS in rapid and high-throughput prediction of physico-chemical properties and selection of internal standards during bioanalytical method development of NMEs**

Mayatra SJ, Sharma D, Prasad B, Singh S

***NIPER, S.A.S Nagar, Mohali-160062, Punjab, India.***

A sensitive and high-throughput method was developed for prediction of critical physicochemical properties (*i.e.,* solubility and Log P value) and selection of internal standards for bioanalysis of new molecular entities (NMEs) during early metabolism and pharmacokinetic studies. The method involved use of liquid chromatography-mass spectrometry (MicrOTOF-Q, Bruker Daltonics, Bremen, Germany), where buffer (pH 7.0) and acetonitrile were used in the mobile phase, whereas C-18 column (Zorbax, 250×4.6 mm, 5 *µ*, Agilent Technologies, USA) was used as a stationary phase. A wide variety of commercially available drugs (~36) were selected as standards. A mixture containing 25 *µ*g/ml each was injected (3*µ*l) into the LC-MS at a flow rate of 0.8 ml/ min in gradient mode for 110 min. In another chromatographic run, a solution containing the same concentration of 6 NMEs from quinolone series was injected. The data were acquired using both UV (at 254 nm) and MS detectors (at optimized parameters). The retention times (RTs) of the standard set of drugs were determined from extracted MS ion chromatograms. Standard curves were plotted using RT and experimental solubility/Log P values. The calibration equations were derived from the curves wherefrom solubility and Log P values were predicted for the test NMEs. The experimental data of test quinolones very well correlated to the predicted values. Another use envisaged of the study is selection of internal standard during bioanalytical method development for NMEs, which can easily be accomplished based on comparison of RTs of the standards and NMEs. Considering the scarcity of amount of the NMEs during early discovery stages, the proposed strategy can be useful for the suggested purposes.

**197**

**Disposition of RBx 2258, a novel alpha 1A adrenoceptor antagonist in rat and dog**

Kakar S, Arora S, N Anand

***Ranbaxy Research Laboratories, Gurgaon, India.***

**Objective:**

To study disposition of RBx 2258, an alpha 1A adrenoceptor antagonist during preclinical development.

**Methods:**

The pharmacokinetics of RBx 2258 was investigated in Wistar rat and Beagle dog following oral and intravenous administration of 5mg/kg and 2mg/kg dose respectively. *In vitro* metabolism was studied in human hepatocytes, liver microsomes (rat, dog and human), human S9 fractions, and purified CYP P450s. The excretion mass balance was studied following oral (47.19 *µ*Ci/ kg) and intravenous (24.61 microCi/kg) administration of 1mg/ kg radiolabelled ^14^C RBx 2258 to male Wistar rat.

**Results:**

The absolute bioavailability in rat (1.6 %) was poor as compared to dog (54.5%). The terminal half-life was 0.76 h in rat whereas 0.92 h in dog. Volume of distribution was 3.3 L/kg and clearance 3.0 L/h/kg in rat as compared to dog with volume of distribution 1.5 L/kg and clearance 1.1 L/h/kg. Major metabolite M1 found in human hepatocytes and microsomes was formed by non-enzymatic reaction while M3 was formed by O-demethylation in animal liver microsomes. Inhibition and induction studies indicated the role of P450 2D6 and P450 3A4 in the metabolism of RBx 2258, whereas involvement of P450 1A2 was ruled out. 85-90% of the radioactivity was recovered in urine and faeces within 96 h.

**Conclusion:**

Oral PK of RBx 2258 in rat and dog indicated its rapid absorption and clearance. The *in vitro* metabolic profiles in rat, dog and human were similar. RBx 2258 is cleared rapidly within 96h in the form of metabolites via urine and faeces.

**198**

**Modulation of P-glycoprotein and cytochrome P450 (CYP3A4) by extracts of *Turnera diffusa* leaves**

Sinha SD^1^, Harle UN^1^, Gaikwad NJ^2^

***^1^AISSMS College of Pharmacy, Kennedy Road, Pune-411001; ^2^Department of Pharmaceutical Sciences, R.T.M. Nagpur University, Nagpur-440033, India.***

Herb-drug interactions are evidenced by many preclinical and clinical case reports resulting in precipitating fatal toxicity. Many pharmacokinetic herb-drug interactions have explored cytochrome 450 enzymes and P-glycoprotein (Pgp) as the prime targets common for herbal drugs and therapeutic medicinal agents. *Turnera diffusa* is well known herb with verity of pharmacological actions like antispasmodic, diuretic, laxative, stomachic and reported to interact with some drugs. The effect of *Turnera diffusa* leaves extract on *in vitro* intestinal permeability of dexamethazone and oral clearance of different probes of cytochrome P450 was studied. Acute and chronic treatment of *Turnera diffusa* significantly (*P* < 0.01, F = 4.58, Df = 4) increased the absorptive permeability of Dexamethazone as compared with that of saline and found even more than that of verapamil pre-treated groups and there by showed inhibition of Pgp. In studying the effect of *Turnera diffusa* extracts on metabolizing enzymes CYP 450; CYP1A2, CYP2C9, CYP2D6, are found to be not affected, but CYP3A, were found to be significant inhibited by hydroalcoholic extracts of *Turnera diffusa*. It may conclude that *Turnera diffusa* showed inhibition of Pgp and CYP3A indicates its potential for modulation of absorption and metabolism of therapeutic drugs and should be further evaluated for pharmacokinetic herb-drug interaction.

**199**

**Antioxidant activity (AOA) of nonsteroidal anti–inflammatory drugs (NSAID) *in vitro***

Mehrotra A, Pant KK, Ali B

***Chhatrapati Shahuji Maharaj Medical University, Lucknow, India.***

Free radicals are unstable and highly reactive species that cause membrane lipid peroxidation, protein denaturation, DNA strand break, and mitochondrial and lysosomal lysis leading to cell injury. There is now substantial evidence that oxidative stress plays an important role in the inflammatory diseases. The aim of the present study was to elucidate the antioxidant activity of five non steroidal anti-inflammatory drugs, namely acetylsalicylic acid, sodium salicylate, paracetamol, piroxicam and diclofenac *in vitro*. The spectrophotometric method for determination of anti-inflammatory activity used in our study was based on the principle that Fe-EDTA complex reaction with H2O2 (Fenton reaction) generates hydroxyl radical (OH^.^), which degrades benzoate resulting in the formation of thiobarbituric acid reactive substances (TBARS). The inhibitory potential of the drug against TBARS production was determined as antioxidant activity using uric acid as the standard antioxidant. All NSAID examined in the present study were found to be have antioxidant activity. The I50 value (the drug concentration causing 50% inhibition) of each drug was calculated and found to be in the range of 0.13 - 0.7 mM. Out of these five drugs paracetamol was found to be most active antioxidant. These findings suggest that antioxidant activity contributes to the mechanism of action of anti-inflammatory drugs.

**200**

**A sensitive bio-analytical LC-MS method for determination of curcuminoids in rat plasma**

Sharma SC, Sachin BS, Vandhna B, Meenakshi K, Tikoo MK, Tikoo AK, Khajuria RK, Johri RK

***Indian Institute of Integrative Medicine, Jammu-Tawi, India.***

Currently curcuminoids (Curcumin, Desmethoxy curcumin and Bisdesmethoxy curcumin) are increasingly being consumed as dietary supplement as well as prescribed for various indications as complimentary medicine. In order to evaluate therapeutic efficacy of curcuminoids, it seems important to determine the curcuminoid content in the various biometrics like plasma. In the present paper, we report a sensitive and reproducible method for curcuminoids determination by LC-MS. The mobile phase was a combination of acetonitrile and water (gradient elution) at a flow rate of 0.6 ml/min. The column used was RP-18, 150 × 4mm with 30°C oven temperature. The mass spectrometer was set at negative mode along with ESI source. The total run time was 15 min. The retention time of curcumin, desmethoxy curcumin and bisdesmethoxy curcumin was 10.66, 10.70 and 9.57 min respectively and the lower limit of quantification was 12.5, 12.5 and 25 ppb respectively. The calibration curve was linear over 12.5 to 300 ppb with r^2^ more than 0.999. The solid phase extraction (SPE) technique was used for the recovery of all the three analytes from rat plasma which showed the mean recovery of 74-80% from spiked plasma at three different levels by using SPE. The method was validated in terms of recovery, specificity, accuracy, precision, linearity, limit of quantification and stability. The method was used successfully for simultaneous estimation of all the three analytes in a single run from *in-vivo* plasma samples after oral administration of the same. In conclusion, the accurate, precise and simultaneous determination of various curcuminoids would find potential application in pharmacokinetic/ pharmacodynamic studies.

**201**

**Improvement of oral bioavailability of etoposide by 4-Ethyl 5-(3, 4 methylenedioxyphenyl)-2e, 4epentadienoic acid piperidide (SK-20)**

Sachin BS, Najar IA, Vandhna B, K Meenakshi, Tikoo MK, Tikoo AK, Sharma SC, Koul JL, Koul SK, Johri RK

***Indian Institute of Integrative Medicine (CSIR) (Formerly RRL), Jammu, India.***

Low oral bioavailability of important drugs, which are used perorally, remains an area of major clinical concern. Extensive research has been conducted at IIIM, Jammu, to address the problem of poor/ variable bioavailability of clinically important drugs including anti-cancer drugs. Etoposide is one such highly prescribed drug for the treatment of wide spectrum of human cancers. Etoposide presents incomplete and variable bioavailability after oral dosing and in most cases it is used as a high-dose intravenous infusion. In recent times a new class of compounds has been synthesized and evaluated, in our Institute, as bioavailability enhancers for a variety of drugs in diverse categories. One such compound, SK-20, has shown significant enhancement in plasma levels of etoposide. The concentration-time curves for the etoposide (20 mg/ kg, po) alone and etoposide in combination with SK-20 (20 mg/kg, po) were established in mice. The bioavailability indices (AUC, Cmax, Tmax) were determined pharmacokinetically by a non-compartmental analysis. The results showed that SK-20 enhanced Cmax of etoposide by 80% and AUC (0-24 hrs) by 52%, whereas the Tmax remained same (0.5 hrs). Several mechanisms for the possible bioavailability enhancement were explored. These included (a) permeability alterations using everted gut sac and Parallel Artificial Membrane Permeability Assay (PAMPA) (b) Phase I metabolic modification via microsomal CYP450 enzymes including 3A4 and, (c) P-gp mediated efflux regulation using in–situ single pass perfusion model. The potential role of bioenhancers to modify the oral bioavailability of drugs has been discussed in context of overcoming pharmacokinetic disadvantages and toxicity concerns of important anti-cancer drugs.

**202**

**Green tea with pioglitazone is pharmacokineticaly safe in spite of their simultaneous interaction with CYP3A**

Gawali NB, Bansod KU, Dixit PV, Umathe SN

***Department of Pharmaceutical Sciences, Rashtrasant Tukadoji Maharaj Nagpur University, Mahatma Jyotiba Fuley Shaikshanik Parisar, Amravati Road, Nagpur – 440 033, (India).***

**Objective:**

Using herbal preparation along with antidiabetic agents is a very common practice in diabetic patients, without any consideration to the possible interaction among them. Green tea is one such herbal preparation, which inhibits, cytochrome P450, a family of enzymes that metabolizes pioglitazone—a commonly used oral antidiabetic agent. Hence, the influence of green tea on the pharmacokinetics of pioglitazone has been investigated in rats to assess the safety of this combination.

**Materials and Methods:**

After the approval by IAEC, male Sprague Dawley rats (180-300g) received green tea extract (GTE) (10mg/kg) and at the end of the experiment its influence on CYP3A activity was investigated *in vitro* using erythromycin-N-demethylase assay employing rat intestinal and liver microsomes. In another set of experiment pioglitazone was administered orally (10mg/kg)/intravenously (5mg/kg) to a group of rats that received green tea or vehicle orally. The blood levels of pioglitazone were estimated using HPLC.

**Results:**

The *in vitro* studies indicated that GTE inhibits the CYP3A activity in intestinal microsomes with no influence in liver microsomes. *In vivo* studies indicated that GTE administration inhibited CYP3A activity in intestine, and activated the same in liver. The kinetic studies revealed that GTE had no influence on the blood levels of pioglitazone, indicating that the increased metabolism in liver is probably compensated by decreased metabolism at intestine level during absorption.

**Conclusion:**

In spite of the modulation of CYP3A activity by GTE, its use along with pioglitazone appears therapeutically safe as the blood levels of pioglitazone remains unaffected.

**203**

**Development and evaluation of an acute experimental model for postmenopausal dry eye syndrome**

Smrita, Velpandian T, Sharma N, Titiyal JS, Biswas NR, Ghose S

***Department of Ocular Pharmacology and Pharmacy, Dr.R.P.Centre for Ophthalmic Sciences, All India Institute of Medical Sciences, Ansari Nagar, New Delhi – 110029, India.***

**Purpose:** To develop and evaluate an acute experimental model for dry eye syndrome in female rats.

**Methods:**

Wister female rats (n=5) weighing 100-200g were used. All female rats were orally administered finasteride 1.16mg/Kg along with acacia for 11 days. The baseline tear flow assessment and tear breakup time were performed using cotton thread technique and slit lamp ophthalmoscope for 6 days alternatively before oral drug administration. Phenol red coated cotton thread was validated for length and diameter for the calculation of volume of tear/ min. The angle of contact of the cotton thread was made acute at the site of lateral canthus. On 12th and 13th day tear flow assessment and tear break up time was reperformed. For all the days, animals were kept under controlled conditions of temperature and humidity to avoid any change in tear flow due to the variation in the ambience.

**Results:**

The basal tear flow values and tear break up time before and after oral administration were found to be 8.28 ± 1.34 mm: 42.5 ± 2.89 sec and 5.94 ± 1.34 mm: 14.6 ± 0.0 sec respectively. The percentage decrease of tear flow and tear break up time were observed as 28.26% and 65.64%.

**Conclusion:**

The Oral finasteride administration significantly decreased the tear film secretion and tear breakup time. This model is under further evaluation for its suitability for postmenopausal dry eye syndrome.

**204**

**Chronopharmacokinetics of cardiovascular drugs - a study**

Usha YN, Mutalik S, Gupta PD, Udupa N

***Manipal College of Pharmaceutical Sciences, Manipal University, India - 576104.***

Chronopharmacokinetics deals with the study of sequential changes in absorption, distribution, metabolism and elimination, which may vary with time of day. Time of day has to be regarded as an additional variable influencing the kinetics of a drug. New formulations with constant or programmable delivery rates now make it possible to deliver a drug at a definite time or at a controlled rate in chronokinetic studies. These are useful to control the diseases which show symptoms at particular time. In the majority of individuals - normotensives and hypertensive blood pressure rises rapidly in the early morning hours, the time when most individuals wake up and begin their day, which may lead to serious cardiovascular complications. For this reason a capsule-type dosage form of valsartan was developed for controlled delivery. The system was designed by imparting a timed-release function to a hard gelatin capsule. The technical characteristics of the system are to contain a swellable polymer together with a table containing active ingredient and erodable tablet in a capsule coated with water insoluble polymer. In order to find the suitable formulation, various formulation factors were investigated through a series of in vitro dissolution studies. As a result, we found the amount of swellable polymer and weight of erodable tablet influenced the controlled release of drug. This result suggested that this approach can provide a useful means for timed release of drug and may helpful for patients with morning surge.

**205**

**Preclinical pharmacokinetics and metabolism of a novel muscarinic receptor antagonist, 33B4088C in rat and dog**

Sharma P, Ahmed T, Singh TP, Varshney B, Gautam A, Paliwal JK

***Ranbaxy Research Laboratories, Gurgaon, India.***

**Introduction:**

33B4088C is a novel muscarinic receptor antagonist synthesized for the treatment of overactive bladder and urinary incontinence. ADME of this molecule was evaluated during lead optimization phase to ensure drug ability.

**Method:**

Pharmacokinetics of 33B4088C was investigated in rat and dog after oral and intravenous administration. The intrinsic clearance was determined in rat, dog and human liver microsomes. The CYP inhibition assay (IC_50_) was performed using human recombinant CYPs *viz*, CYPIA2, 2C9, 2C19, 2D6, and 3A4. The permeability was determined in Caco-2 cells and the plasma protein binding was evaluated by equilibrium dialysis using rat and human plasma.

**Results:**

Absolute bioavailability in rats was 28% with a clearance of 4.17 L/hr/kg and volume of distribution of 11.02 L/ kg. An absolute bioavailability of 83% was observed in dogs with a clearance of 0.49 L/hr/kg and a volume of distribution of 4.28 L/ kg. The in-vitro intrinsic clearance was low in all species. The CYP inhibitions (IC50) were greater than 10*µ*M for all CYPs indicating unlikeliness of CYP mediated interaction in man. 33B4088C exhibited moderate permeability and low plasma protein binding in rats (26%) and human plasma (40%).

**Conclusions:**

33B4088C was metabolically stable in liver microsomes with no CYP inhibition liability in human. It had good oral bioavailability in dog. Based on *in-vitro* and *in-vivo* studies it can be concluded that 33B4088Cis a druggable molecule

**206**

**Development and validation of sensitive ELISA method for quantification of filgrastim (RHG-CSF) in human serum: Application to a pharmacokinetic study**

Agarwal N, Jain S, Behl V, Varshney B, Paliwal JK

***Ranbaxy Research Laboratories, Gurgaon, India.***

**Introduction:**

Filgrastim is a 175 amino acid protein produced by a strain of *Escherichia coli* bearing a genetically engineered plasmid that contains a human granulocyte colony-stimulating factor (G-CSF). It has biological actions similar to that of endogenous G-CSF.

**Method:**

In the present work a sensitive, accurate and precise enzyme linked immunosorbent assay for quantitation of G-CSF in human serum was developed and validated using Instant ELISA kit of Bender Med system. The validation includes assessments of method for precision and accuracy, specificity, recovery, matrix effect, dilution integrity and stability in human serum. The validated method was used to analyze samples from an open label, single-treatment, twoperiod, single-dose, and replicate design pharmacokinetic study, conducted to study the intrasubject variability in pharmacokinetic parameters of Filgrastim following single dose administration through subcutaneous route in healthy, adult, human, male subjects.

**Result:**

Acceptable precision and accuracy (± 25% deviation for LLOQ and ULOQ standards and ± 20% deviation for other standards from the respective nominal concentration) were obtained for concentrations over the standard curve ranges. Statistical analysis was performed on the completed subjects. 95% confidence interval for the ratio of the product averages in Period I (P_1_) and Period II (P_2_) was calculated for C_max_ as 109.60%, AUC_0-t_ as 110.05% and AUC_0-inf_  as 111.22%.

**Conclusions:**

The validated method was found to be precise and accurate. Intrasubject variability was below 20% and a BE study would typically require 26 subjects.

**207**

**Quantitative determination of main monohydroxylated products of stanozolol by high performance liquid chromatography-tandem mass spectrometry **

Ahi S, Beotra A, Jain S

***National Dope Testing Laboratory, Ministry of Youth affairs and Sports, New Delhi, India.***

**Introduction:**

Stanozolol is one of the most abused anabolic steroid in sports in India. Analysis of this compound is very challenging as it must be detected and confirmed at Minimum required performance limit (MRPL) of 2ng/ml as set by World Anti Doping Agency (WADA). To determine very low levels of main mono-hydroxylated products of stanozolol viz. 3-OH-Stanozolol, 4-B-OH-Stanozolol and 16-B-OH-Stanozolol in human urine an analytical method was developed and validated by the use of Liquid Chromatography Tandem Mass spectrometry (LC-MS/MS) technique.

**Materials and Methods:**

The urine samples were spiked in 5 replicates with 3-OH-Stanozolol, 4-B-OH-Stanozolol and 16-B-OH-Stanozolol in different concentration of 1, 2, 4 and 10ng/ml, were processed by Liquid-Liquid extraction procedure and were then injected on LC-MS/MS system. The method validation was done as per WADA International Standard for laboratories guidelines keeping in view linearity, precision, accuracy, specificity and recovery.

**Result and Discussion:**

The calibration curve was found linear in the range from 1-10ng/ml for all the three metabolites with coefficient of correlation achieving to a degree of R=0.99. The recoveries of all analytes were reported to fall between 80-100%. The Limit of detection was found to be 0.5ng/ml and the Limit of Quantitation was reported to be 1ng/ml. Pharmacokinetic profile of the drug administered orally shows that 3-OH-Stanozolol is found at highest concentration, followed by 4-B-OH-Stanozolol and the least excreted was 16-B-OH-Stanozolol. Based on the study and results the method was applied to 63 old doping control samples which were previously tested positive for stanozolol misuse which too showed the same pattern of metabolite profiling.

**Conclusions:**

It can thus be concluded that with its low quantitation limit using LC-MS/MS, 3-OH-Stanozolol can be treated as a long term and effective marker for stanozolol abuse.

**208**

**A novel drug delivery approach to reduce dose related side effects of acyclovir – a BCS class III drug**

Halim A, Chakraborty T, Halder S

**Krupanidhi College of Pharmacy, Bangalore, India.**

Acyclovir, a very potent antiviral drug listed in the top 200 drugs in USA is a BCS class III drug, acyclovir has very low bioavailability (10-20%) due to its low intestinal permeability, though it has high solubility still it requires a very high dose (400-800mg) to obtained desire pharmacological effect. Because of the high dose of therapy it produce several dose related side effect which include- Agitation, Confusion, Encephalopathic changes, Hemolytic uremic syndrome, Lethargy, Encephalopathic changes, Renal impairment, Thrombotic thrombocytopenic purpura, Immunocompromised patients even some fatal cases observed. To overcome these complication the present study was made to improve oral bioavailability of acyclovir by formulating w/o type microemulsion using different types of vegetable oil (cotton seed oil, sesame oil, olive oil etc), surfactant (span20, span40, span60, span80) and co-surfactant (ethanol, glycerol, PEG etc). The optimized formulations were studied pharmacologically to check the improve in intestinal permeability by everted sac method in isolated intestinal tissue of wister rats. The pharmacological studies showed marked improve in intestinal absorption with respect to solutions, emulsion, capsule and tablet of acyclovir. Based on above study it was concluded that the oral microemulsion system has the capability to enhance the drug permeability and hence increase bioavailability of acyclovir (BCS class III drugs). Therefore it can be inferred that this attempt reduced the intake amount of the acyclovir and hence decrease the dose related side effects.

**209**

**Preclinical *in vitro* and *in vivo* disposition of an antimalarial compound piperaquine**

Gigras R, Gautam A, Paliwal JK

***Ranbaxy Research Laboratories, Gurgaon, India.***

**Introduction:**

Piperaquine is an antimalarial being used as a partner with short acting antimalarials. Preclinical ADME was determined prior to safety evaluation in experimental animals and results are reported.

**Method:**

Metabolic stability of Piperaquine was assessed in liver microsomes of various species. The CYP inhibition potential and reaction phenotyping was determined in purified Cytochrome P450s. *In-vitro* plasma protein binding and blood to plasma partitioning was determined in rat, mouse, dog and human. Absolute oral bioavailability was studied in rat and mouse at 5mg/kg intravenous and 25mg/kg oral administration. Pharmacokinetic parameters were also determined in dog (10mg/kg p.o) and monkey (5, 25 and 50mg/kg p.o).

**Results:**

The degradation rate constant of Piperaquine was least in rat <dog<mouse=human<monkey liver microsomes. CYP3A4 was the major enzyme involved in the metabolism of Piperaquine. Piperaquine inhibited CYP3A4 with an IC_50_ of 5*µ*M. The plasma protein binding was >99% in all species tested and the blood to plasma ratio was ~1. In rat and mouse Piperaquine showed good oral bioavailability (60%), long terminal half life and large volume of distribution. Systemic clearance of Piperaquine is higher in rat than mouse. Dog displayed a long oral elimination half life. In monkeys the oral clearance was high and the exposure was least.

**Conclusions:**

Piperaquine is metabolically stable in liver microsomes of all species. It is metabolized mainly by CYP3A4 and also inhibits CYP3A4. Oral bioavailability is good in rat and mouse with a very long half life. Dog shows a long oral half life and good exposure whereas the exposure was the least in monkey.

**210**

**Characterization and structure elucidation of heme adduct of a novel anti-malarial drug - arterolane (RBX11160) maleate**

Jaggi S, Varshney B, Singh Y, Jain G, Srinivas KS, Paliwal JK

***Ranbaxy Research Laboratories, Gurgaon, India.***

**Introduction:**

Arterolane (RBx 11160) Maleate – the novel synthetic endoperoxide antimalarial had been reported to be highly active against *P.falciparum*. Being an endoperoxide we hypothesize that the mechanism of action involves the intracellular reduction of the peroxide group and the subsequent generation of Carbon-4 centered reactive species. The reactive species may give rise to specific parasite toxicity by inducing oxidative stress resulting in apoptosis, alkylation of parasite proteins, or alkylation of heme by forming adducts. In the present study, using LC/MS and NMR experiments an attempt has been made to provide experimental evidence for the hypothesis of Carbon-4 centered reactive species generation.

**Methods:**

RBx11160 was incubated *in vitro* with hemin to yield RBx11160 – heme adducts with formation of alkylated products at four meso positions. The heme-RBx11160 adducts were isolated from the reaction mixture by preparative reverse phase HPLC on C18 column. A gradient of 0.1% triflouroacetic acid in HPLC grade water and acetonitrile was employed to purify the heme-RBx11160 adduct. The fraction collected was then lyophilized to get solid dried heme-RBx11160 adduct. The structure of heme-RBx11160 adduct was characterized by ‘H NMR Spectroscopy.

**Results:**

LC/MS analysis revealed four peaks of identical masses and heme like spectra. Fraction collection, enrichment using semi preparative HPLC and NMR analysis of the most intensive peak enabled us to elucidate mechanism of adduct formation and the definitive structure of adduct.

**Conclusion:**

LC/MS and NMR techniques were utilized to identify four adducts at alpha, beta, gamma and delta positions of heme.

**211**

**Comparative efficacy and safety of various antimicrobialsin patients of acute rhinosinusitis at tertiary-care hospital**

Sharma Vivek, Mishra KC^4^, Saxena RK^3^, Sharma Geetanjali^2^, Sharma Shalini^1^

***Dept. of Pharmacology, Physiology^2^, Pt BDS University of Health Sciences, Rohtak.(HR), Dept. of Pharmacology^4^, ENT^3^, Physiology^1^, HIMS, Dehradun (U.K).***

**Objective:**

(1) To compare the efficacy of amoxicillin, amoxicillin plus metronidazole and azithromycin for the treatment of Acute Rhinosinusitis (ARS). (2) To monitor adverse drug reaction profile of amoxicillin, amoxicillin plus metronidazole and azithromycin in patient with ARS.

**Materials and Methods:**

An open randomized trial of comparative efficacy and safety of amoxicillin, amoxicillin plus metronidazole and azithromycin in patients with ARS. Patients were randomized into three groups as under:-Group 1 – Patients on oral amoxicillin 500mg TDS for 10 days; Group 2- Patients on oral amoxicillin 500mg TDS for 10 days plus oral metronidazole 400mg TDS for 10 days. Group 3 – Patients on tablet azithromycin 500mg OD for 5 days. Patients were evaluated for signs and symptoms at Day 1, Day 7 (for Group 3), on Day 12 (for Group 1 and 2) as primary end points and 28 days after post therapy as secondary end point.

**Results:**

All the three antimicrobial drugs i.e. amoxicillin and amoxicillin plus metronidazole and azithromycin were effective in reducing symptoms of acute sinusitis on Visual analogue scale (VAS) scoring. Combination of amoxicillin plus metronidazole was more effective than azithromycin in reducing symptoms of acute sinusitis on day 40(*P*<0.001 Vs *P*<.01). Azithromycin showed significant improvement in radiographic scoring on day 7 (*P*<0.01) and on day 35 (*P*<0.01).Amoxicillin plus metronidazole when compared with amoxicillin showed significant improvement in radiographic scoring both on Day12 (*P*<0.05) and Day 40 (*P*<0.001). Amoxicillin was associated with maximum adverse effects. Metronidazole was associated with metallic taste, whereas azithromycin was associated with least adverse effects.

**Conclusion:**

All the three antimicrobial drugs i.e. azithromycin, amoxicillin and amoxicillin plus metronidazole were effective in reducing symptoms of acute sinusitis on visual analogue scale (VAS) scoring. But combination of amoxicillin with metronidazole was associated with earliest improvement in radiographic scoring.

**212**

**Pharmacokinetic interaction of *Tinospora cordifolia* with some therapeutic medicinal agents**

Bajaj AO^1^, Harle UN^1^, Gaikwad NJ^2^

***^1^AISSMS College of Pharmacy, Kennedy Road, Pune-411001; ^2^Department of Pharmaceutical Sciences, RTM- Nagpur University, Nagpur-440030, India.***

*Tinospora cordifolia* is widely used in traditional system of medicine to treat jaundice, rheumatism, urinary diseases, jaundice, intermittent fever and eye aliments. It is reported for antipyretic, alterative, diuretic, anti-inflammatory and antirheumatic activities. With the preliminary reports on herb-drug interaction *Tinospora cordifolia* was studied for pharmacokinetic interaction with some therapeutic medicinal agents like warfarin, furosemide and diazepam. In this study, 500 mg/kg/day (po) *Tinospora cordifolia* extract was given for five days as an oral suspension and the last dose was coadministered with therapeutic dose of warfarin, furosemide and diazepam in rats. The blood samples were collected following the co-administration of *Tinospora cordifolia* and therapeutic medicinal agents and drug was extracted in organic solvents and processed for bioanalysis using standardized quantitative High Performancy Liquid Chromatographic method and pharmacokinetic evaluation was performed. Chronic administration of *Tinospora cordifolia* 500 mg/kg/day significantly decrease the plasma concentration of warfarin, furosemide and diazepam in rats. It may be concluded that the *Tinospora cordifolia* should be contraindicated with warfarin, furosemide and diazepam and further it should be studied for pharmacokinetic interaction with other therapeutic medicinal drugs.

